# Diffuse Bilateral Multifocal Bone Stress Injuries in a 19-Year-Old Male Military Trainee: A Case Report

**DOI:** 10.7759/cureus.117025

**Published:** 2026-09-27

**Authors:** Anthony L Carbone, Paul J Wurtz, Courtney H Merseal, Cody R Butler

**Affiliations:** 1 Internal Medicine, Brooke Army Medical Center, Fort Sam Houston, USA; 2 Sports Medicine, San Antonio Uniformed Services Health Education Consortium, San Antonio, USA; 3 Research, San Antonio Uniformed Services Health Education Consortium, San Antonio, USA

**Keywords:** active-duty military personnel, athlete workload, bone stress, bone stress injuries, covid-19, military training, relative energy deficiency in sport

## Abstract

Bone stress injuries (BSIs) are common among high-performance athletes and military trainees. BSIs typically present with point tenderness, loading pain, and nighttime discomfort, and most involve only one or two bones, with clinical symptoms roughly proportional to the degree of osseous injury. We present the case of a 19-year-old male engaged in high-intensity Air Force Special Warfare training who initially presented with vague low back, thigh, and foot pain and was ultimately diagnosed with diffuse multifocal grade 3 and 4a BSIs involving numerous bones of the bilateral lower extremities. What makes this case unusual is the extent and severity of osseous injury relative to the patient’s comparatively mild and nonspecific symptoms. Further, the case demonstrates the complexity of bone stress pathology and highlights the possibility that reduced physical activity during adolescence contributed to later bone fragility.

## Introduction

Injuries to both soft tissue and bone are common among military personnel and athletes. While fractures are often linked to acute traumatic events, researchers have continued to evaluate the impact of repetitive submaximal overloading, which can lead to the microtrauma that underlies bone stress injuries (BSIs) and their potential predisposing factors [1].

The clinical presentation of BSI varies in severity and location and can be influenced more broadly by an individual’s nutrition and physical activity level. Patients often describe an acute increase in physical activity followed by localized tenderness that worsens with activity, aching at rest, and pain exacerbated by direct palpation of bony structures [1,2].

Radiographic imaging is essential for diagnosis. While plain radiographs are typically ordered during preliminary evaluation, higher-order imaging with magnetic resonance imaging (MRI) is considered the gold standard for diagnosing BSI. Understanding and promptly managing BSIs is crucial to maintaining the health and performance of athletes and military personnel [2,3].

BSIs are highly prevalent as orthopedic injuries within athletic populations. Abbott et al. estimated approximately 10% of all orthopedic injuries in athletes are attributed to BSIs, with higher incidence rates amongst female athletes, and further noted BSIs may affect up to 40% of athletes during their careers [4-6]. BSI incidence among trainees has been studied extensively in the US military. During a 10-week Army basic training period, the reported occurrence rate was 20 per 1,000 male trainees and 80 per 1,000 female trainees [7,8]. Even in more physically demanding military training environments, similar rates of injury were noted among Air Force Special Warfare Trainees: 122 of 2,156 male trainees and two of 10 female trainees [4]. BSIs have been shown to negatively affect trainee advancement and healthcare utilization [7,8].

A systematic review of modifiable and nonmodifiable risk factors for BSIs in military trainees identified several key findings. Nonmodifiable risk factors included older age, prior stress fracture, female sex, menstrual dysfunction, and race other than Black [1]. Modifiable risk factors such as nutrition, body mass index (BMI), fitness level, and muscle strength were also identified as contributors to BSI susceptibility [4]. Understanding these risk factors is crucial for effective prevention and management of BSIs in physically active populations.

When diagnosing a BSI, staging based on the severity of clinical and imaging findings is important. The Fredericson classification system, which relies on MRI findings, offers characterization and scoring to objectively categorize the extent of injury. The classification system itself was originally designed to grade tibial stress injuries but has since been applied more broadly.

Grade 1 injuries demonstrate periosteal edema only, while grade 2 injuries show bone marrow edema visible only on T2-weighted sequences. Grade 3 injuries demonstrate bone marrow edema visible on both T1- and T2-weighted sequences, and grade 4 injuries involve intracortical signal abnormality, further subdivided into grade 4a, characterized by multiple focal areas of intracortical signal abnormality, and grade 4b, characterized by a linear intracortical signal abnormality consistent with a cortical stress fracture. In the modified Fredericson system, the original grade 4 was subdivided into grades 4a and 4b to distinguish focal intracortical signal changes from a discrete cortical fracture line [9].

While staging informs decisions based on severity, other factors significantly influence management. The location of the BSI and the presence of a displaced fracture play a pivotal role, as poor-prognosis locations or displacement may necessitate surgical intervention [7,10]. By far the most common presentations of BSI are grade 2 or lower, with localizable tenderness and radiographic changes limited to one or two bones [11].

With the exception of clear indications for surgery, such as displacement, most BSIs are managed conservatively. The conservative treatment regimen typically involves combinations of rest, analgesia, physical therapy, and a structured, gradual return to activity [1,3,7]. Research has shown that, on average, the time from injury to full return to activity is approximately 12 to 13 weeks [7,10].

Here we present an unusual case of BSI that deviates from typical presentations in both the diffuse distribution and severity of injury: a 19-year-old male military trainee with diffuse bilateral multifocal grade 3 and 4a BSIs.

## Case presentation

A 19-year-old Caucasian male military trainee (80 kg, 178 cm) initially presented with acute low back pain (LBP) during a ruck march. Activities such as rucking, lengthy weighted marches, and other demanding, high-impact activities typified daily training regimens for Air Force Special Warfare training. The patient’s initial evaluation was largely benign, with no significant musculoskeletal abnormalities or sensorineural deficits, and he was diagnosed with back pain secondary to compensation from deconditioned core and hip stabilizers. Given the high frequency of similar injury patterns in this training environment, he was managed conservatively with a physical training profile, rest, and physical therapy, and no additional imaging was obtained at that time. He responded well, was reevaluated after one month, and returned to training at that time with a complete resolution of symptoms.

Six months later, the patient reported a subacute progression of his LBP along with new subacute progressive thigh and foot discomfort and bilateral knee pain. Physical examination was significant for bilateral knee pain and bony tenderness over the femoral condyle and tibial plateau. Plain radiographs of the bilateral knees revealed no osseous abnormalities. Given the patient’s presenting symptoms and physical examination findings, there was still a high clinical suspicion for low-grade BSI, as it is not uncommon for there to be no evidence of BSI on plain film. The patient was again referred to physical therapy. After six weeks of being withheld from training and rehabilitation without improvement, plain films of the bilateral tibiae and fibulae and feet and an MRI of the bilateral lower extremities were ordered. While the plain films again demonstrated no significant injury, the MRI revealed extensive multifocal grade 3 and 4a stress injuries involving bilateral patellae, femurs, tibiae (Figures 1, 2), and nearly all tarsal and metatarsal bones (Figures 3, 4).

**Figure 1:**
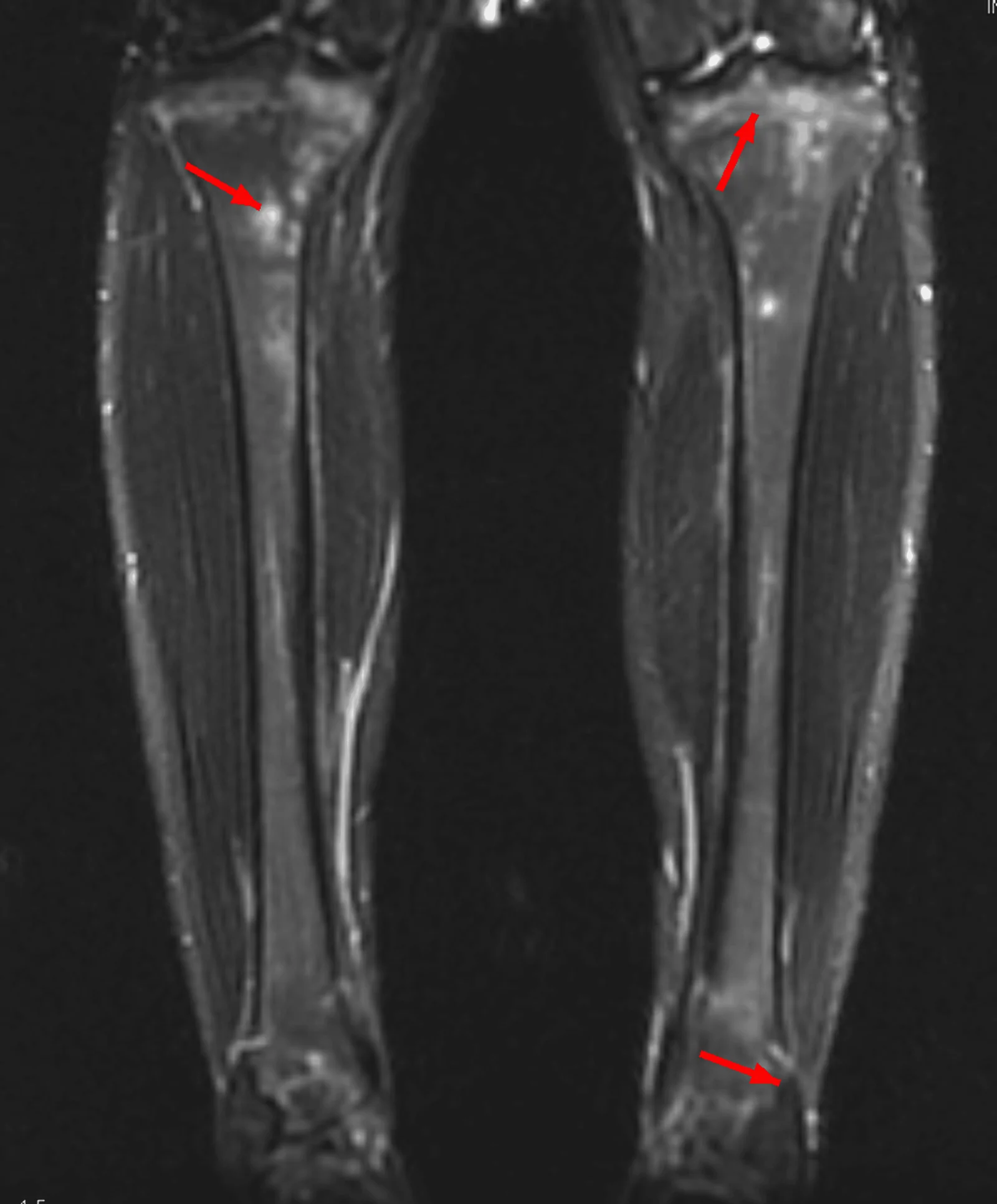
MRI of the tibia and fibula with T1 weighting demonstrating extensive multifocal grade 3 stress injuries of the lower extremities involving the patella, femurs (medial greater than lateral femoral condyles), proximal tibial metaphyses including the tibial plateaus, distal tibial metaphyses including the plafonds, and talar domes, with several regions of severe injury highlighted by arrows.

**Figure 2:**
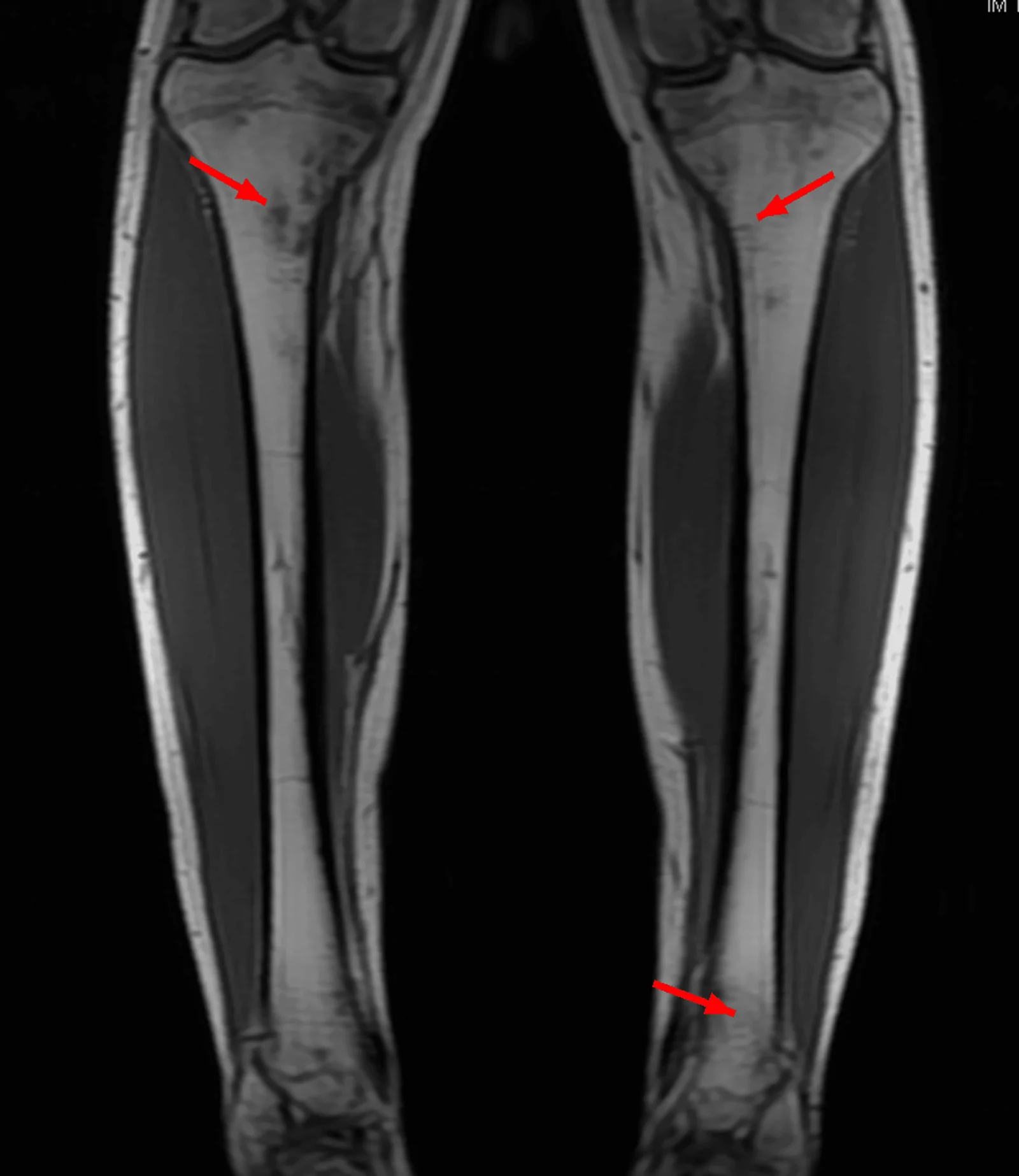
MRI of the tibia and fibula with T2 weighting demonstrating extensive multifocal grade 3 stress injuries of the lower extremities involving the patella, femurs (medial greater than lateral femoral condyles), proximal tibial metaphyses including the tibial plateaus, distal tibial metaphyses including the plafond, and talar domes, with several regions of severe injury highlighted by arrows.

**Figure 3:**
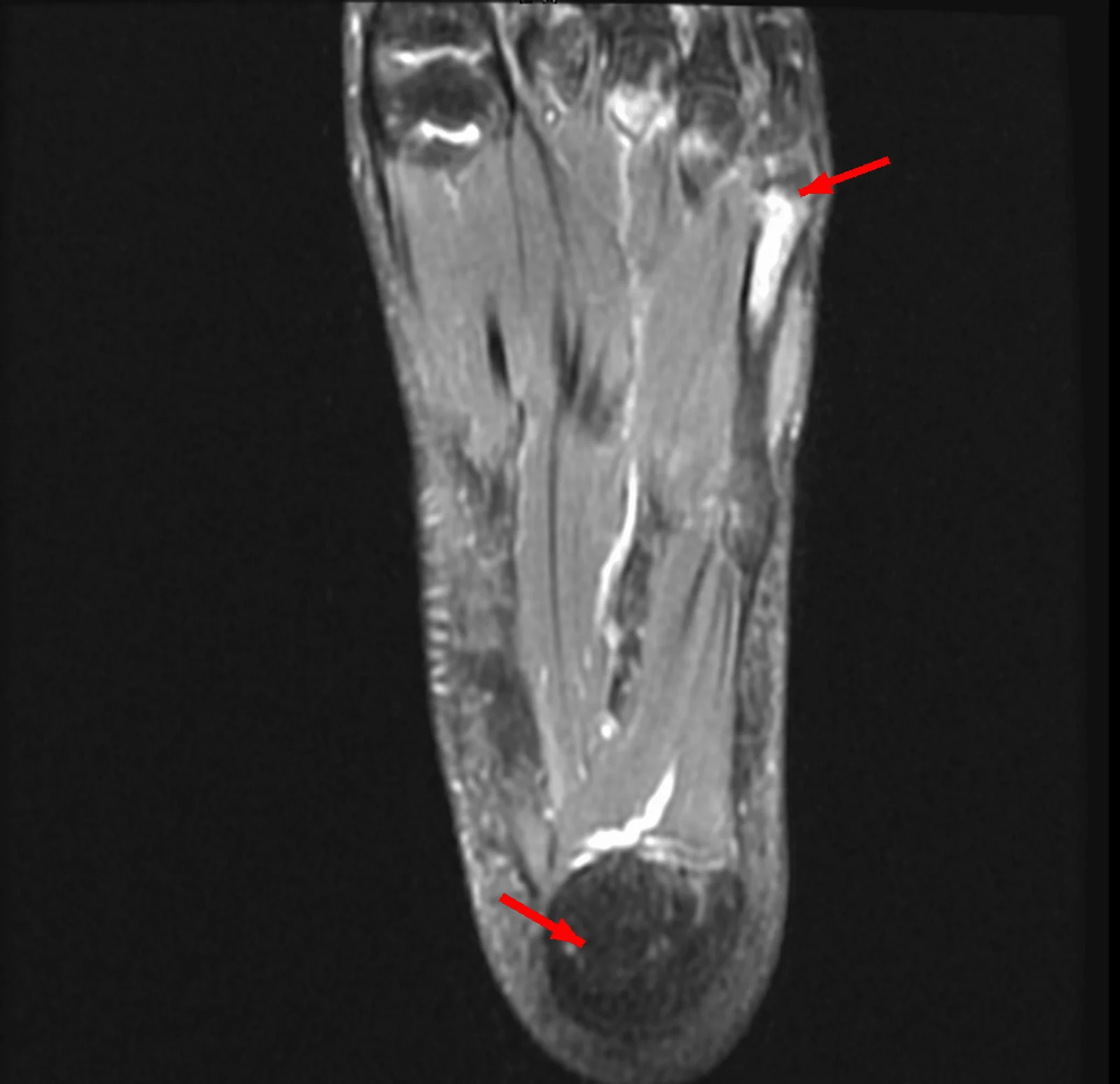
MRI of the left foot without contrast in coronal plane demonstrating extensive multifocal high-grade stress injuries throughout nearly the entire visualized foot, predominantly grade 3 with grade 4 injuries of the talar head and third metatarsal base, with several regions of severe injury highlighted by arrows.

**Figure 4:**
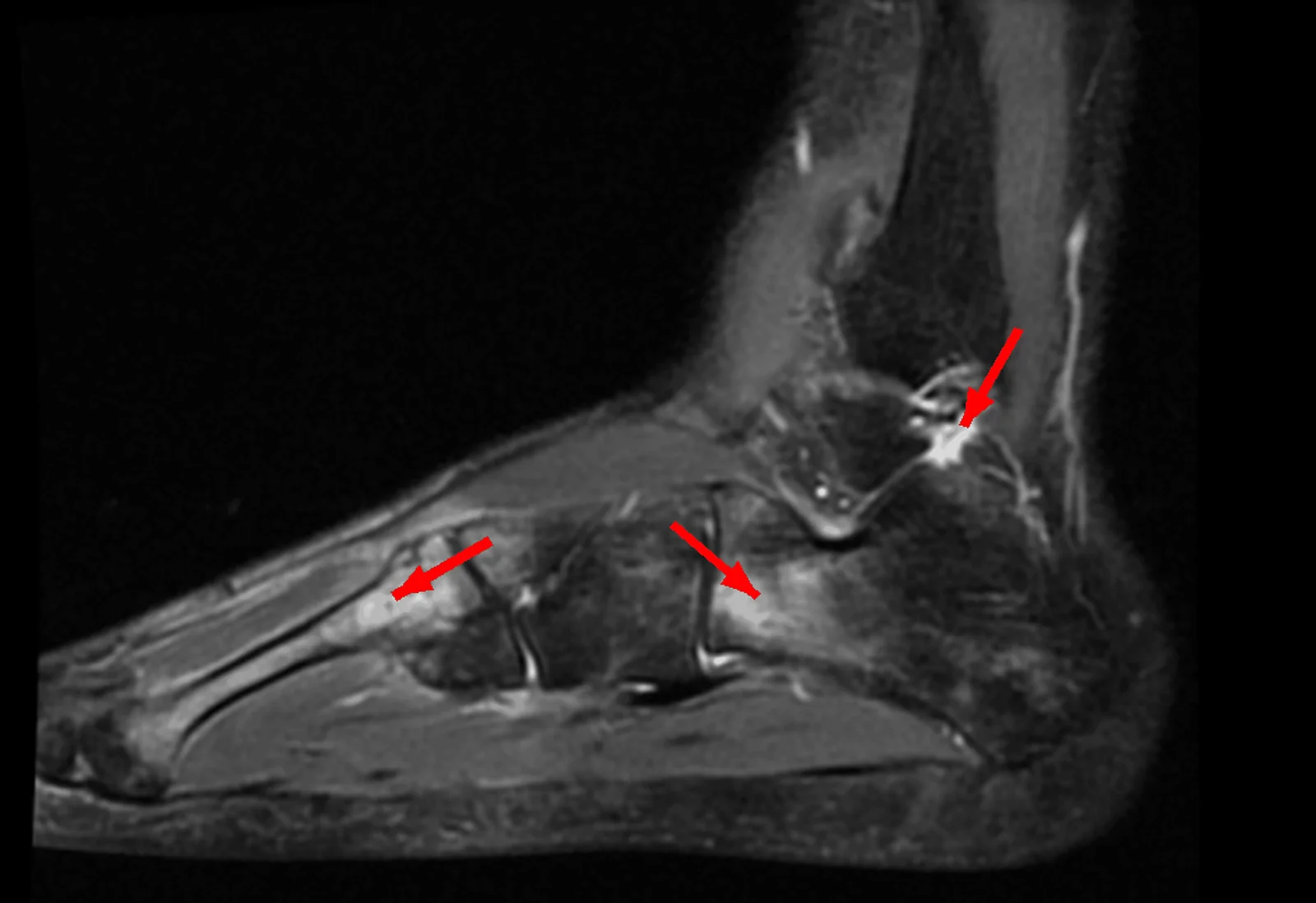
MRI of the left foot without contrast in sagittal plane demonstrating extensive multifocal high-grade stress injuries throughout nearly the entire visualized foot, predominantly grade 3 with grade 4 injuries of the talar head and third metatarsal base, with several regions of severe injury highlighted by arrows.

Diagnosis and treatment

The final diagnosis was established by bilateral lower extremity MRI with injury to bilateral patellae, femurs, and tibiae (Figures 1, 2) and nearly all tarsal and metatarsal bones (Figures 3, 4). Given the extent of the injury, further evaluation of a potential secondary etiology was explored, and an assessment of bone mineralization with dual-energy X-ray absorptiometry (DEXA) revealed Z-scores of -0.5 in the lumbar vertebrae and -1.8 in both the left and right femoral necks (the accepted normal range in the general population is above -2.0; however, this threshold has been shown to underestimate clinically meaningful bone deficits in athletes, as discussed further below). Although a secondary etiology for osteopenia/osteoporosis such as relative energy deficiency in sport (REDs), thyroid, parathyroid, and pituitary abnormalities, or chronic alcohol consumption would more likely produce Z-scores below -2.0, a comprehensive laboratory evaluation for metabolic and hormonal abnormalities was performed given the extent of injury [12]. The workup included a complete blood count (CBC), comprehensive metabolic panel (CMP), thyroid-stimulating hormone (TSH), free triiodothyronine (FT3), parathyroid hormone (PTH), vitamin D level, and testosterone level. These results revealed no specific underlying metabolic or hormonal condition to explain the low bone mineral density; all values were within normal limits, and the vitamin D level was 40 ng/mL (reference range, 30-100 ng/mL). A review of the patient’s medical and social history was not significant for pertinent familial factors, pediatric fractures, growth or developmental delay, illicit substance or alcohol use, or hormonal supplementation. Interestingly, the patient did report approximately three years of minimal physical activity during the COVID-19 pandemic, during which he was primarily sedentary at home prior to reporting to basic training.

Following the patient’s diagnosis, the treatment plan involved high-dose vitamin D supplementation at 50,000 international units once weekly for 12 weeks and deloading of the lower extremities with crutches. The patient utilized crutches to maintain partial weight-bearing of the bilateral lower extremities for four weeks while continuing weekly engagement with physical therapy. Following the four weeks of partial weight-bearing, he demonstrated symptom improvement and was transitioned to a BSI return-to-activity protocol focused on gradually increasing lower-extremity strength and loading over 16 weeks. He responded well throughout treatment and was successfully returned to duty without activity limitations after 16 weeks.

## Discussion

While overuse and rapid loading of bone can lead to BSI, the degree of injury observed in this patient was extreme without evidence of a contributing secondary etiology. A review of the patient’s laboratory results revealed no significant abnormalities, and the training workload mirrored that of previous trainees. During the clinical evaluation, common etiologies such as nutritional deficits and hormonal imbalances were excluded as being contributory. While a metabolic etiology was considered amongst other underlying pathologies, the bone remodeling would have been expected to worsen the patient’s condition over time. Investigation of the patient’s co-trainees revealed that while other trainees experienced BSI, none exhibited the extensive involvement seen in this case.

This case represents an exceptionally rare manifestation of severe diffuse BSIs in a physically active Caucasian male. Notably, it deviates from prior studies that have primarily associated BSI risk with sedentary individuals, race other than Black, female sex, prior stress fracture, and age over 24 years [1,2,4].

Perhaps most interesting in this case, in addition to the extent of the BSI, was the patient’s abnormal femoral bone mineral density. In younger individuals, DEXA interpretation relies on Z-scores rather than T-scores [12, 13]. T-scores compare bone mineral density with that of a healthy young adult at peak bone mass and are primarily used to diagnose osteopenia and osteoporosis in older adults. In contrast, Z-scores compare bone mineral density with age- and sex-matched controls and are therefore the preferred metric in adolescents and young adults, including competitive athletes and military trainees [12,13].

In the general population, a Z-score below -2.0 is considered below the expected range for age [14]. However, experts within the field have pushed for more patient-centered application of the score, arguing the threshold may underestimate clinically meaningful deficits, specifically in young athletes [12,14]. Within this subset, higher bone density is generally expected in the setting of habitual weight-bearing activity [5,10]. As a result, even Z-scores below -1.0 warrant further evaluation in athletes and military trainees, as relatively modest reductions in bone mineral density may substantially increase susceptibility to bone stress injury under repetitive mechanical loading [14].

This patient's lumbar spine Z-score was preserved (-0.5), but bilateral femoral Z-scores of -1.8 were clearly abnormal for a healthy 19-year-old military trainee. Although these values do not establish osteoporosis or identify a specific metabolic disorder, they provide objective evidence that the patient may not have achieved expected peak bone mass. In the setting of an otherwise unrevealing metabolic evaluation, the abnormal femoral Z-scores offer a plausible explanation for why this patient developed unusually extensive BSIs despite training conditions similar to those experienced by his peers [12,14].

Upon further exploration, there are several hypotheses that could explain this presentation, including delayed diagnosis in the setting of atypical presenting features potentially leading to worsened outcomes, extreme training conditions, an unidentified preexisting bone abnormality, or inadequate bone development during adolescence [10]. Given the patient’s abnormal femoral Z-scores, the latter hypothesis is a particularly compelling explanation for the clinical presentation.

During one of the patient’s visits, a crucial element of his history emerged. The patient outlined the specific impact of the COVID-19 pandemic and subsequently the impact on his ability to exercise during adolescence. The patient recounted that the pandemic coincided with his transition to high school, and geographically he was living in a densely populated metropolitan area proximal to New York City. Stringent quarantine measures in his home state removed him from all sports and restricted his participation in physical activity throughout most of high school. From ages 13 to 16, the patient rarely left his house, attended virtual school, and spent much of his time playing video games, a trend not dissimilar to the global adolescent activity level found at times during the pandemic [15]. 

These years represent a critical period of skeletal development, during which a substantial proportion of peak adult bone mass is normally acquired [10,11,14]. Mechanical loading through sports and other weight-bearing activities provides an essential stimulus for bone formation during adolescence. It is therefore plausible that prolonged inactivity during this developmental window contributed to the patient's reduced femoral bone density, as reflected by his abnormal Z-scores, thereby increasing his susceptibility to diffuse, high-grade BSIs once exposed to the demands of military training [12,14,15]. Although this relationship cannot be established from a single case, the combination of abnormal femoral Z-scores, a normal metabolic evaluation, and a prolonged history of reduced adolescent physical activity supports this hypothesis.

Initially, this appeared to be an isolated event rather than a trend. However, there had been case reports from late 2023 to the present that described three separate instances of military trainees who sustained catastrophic atraumatic displaced fractures in the setting of asymptomatic bone stress injuries [5]. Similar to the patient we present, these trainees fell within a similar age range (19-21 years) and were reasonably active during their military training. Although causation cannot be inferred without additional investigation, these reports raise the possibility that our patient's presentation is not an isolated occurrence and that disruption of adolescent physical activity during the COVID-19 pandemic may have contributed to skeletal vulnerability in a subset of otherwise healthy young adults [10,12,15].

Taken together, the observations highlight the importance of interpreting Z-scores rather than T-scores when evaluating bone health in young athletes and military trainees. In patients with multifocal, recurrent, or unexpectedly severe BSIs, abnormal Z-scores may provide an early indication of suboptimal peak bone mass despite an otherwise unrevealing metabolic workup. While generalizability is limited in the findings of one case, should multiple isolated cases continue to appear across the literature, future studies may be warranted to determine whether adolescents whose participation in organized sports and regular weight-bearing exercise was interrupted during the COVID-19 pandemic demonstrate persistently lower bone mineral density and an increased incidence of bone stress injuries after returning to high-demand physical activity [4,12,14].

## Conclusions

This case illustrates an unusually severe and diffuse pattern of grade 3 and 4a BSIs in a 19-year-old military trainee whose symptoms were disproportionately mild relative to the extent of osseous injury and whose metabolic and hormonal evaluation was unrevealing. The most notable finding, however, may not be the severity of the BSIs themselves but the patient’s abnormal bilateral femoral Z-scores; although these values do not meet the conventional threshold used to define bone mineral density below the expected range for age, they are contextually concerning in a healthy young athlete and military trainee, in whom Z-scores below -1.0 warrant further evaluation because they may reflect suboptimal peak bone mass and an increased risk of BSI.

While isolated to this case, these findings may plausibly be explained by prolonged interruption of organized sports and weight-bearing activity during adolescence, coinciding with the COVID-19 pandemic, which may have impaired normal bone development and predisposed him to the observed diffuse multifocal BSIs despite an otherwise benign metabolic workup and training conditions similar to his fellow trainees. Though generalizability may be limited at this time, this case may raise questions about whether further investigation is needed into how reductions in adolescent physical activity during the pandemic could have impacted osseous development.
